# The Effect of Canertinib on Sensitivity of Cytotoxic Drugs in Tamoxifen-Resistant Breast Cancer Cells In Vitro

**DOI:** 10.1155/2018/7628734

**Published:** 2018-10-23

**Authors:** Hesham A. M. Gomaa, Asmaa T. Ali, M. Abdel Gabbar, M. A. Kandeil

**Affiliations:** ^1^Pharmacology Department, College of Pharmacy, Jouf University, Sakakah, Saudi Arabia; ^2^Assistant Professor at Biochemistry Department, Faculty of Pharmacy, Nahda University, Beni-Suef, Egypt; ^3^Assistant Lecturer at Biochemistry Department, Faculty of Pharmacy, Nahda University, Beni-Suef, Egypt; ^4^Lecturer at Biochemistry Division, Chemistry Department, Beni-Suef University, Ph.D. in Biochemistry, Beni-Suef, Egypt; ^5^Professor and Head of Biochemistry Department Faculty of Veterinary Medicine, Beni-Suef University, Beni-Suef, Egypt

## Abstract

**Aims and Objectives:**

To investigate and examine the reversal effects of canertinib on the activity of EGFR and tamoxifen resistance in drug-resistant human breast carcinoma cells (MCF-7/TamR).

**Materials and Methods:**

The antiproliferative activity of canertinib alone or in combination with a conventional EGFR-targeting chemotherapies cytotoxic drugs differing in the mechanism(s) of action, such as paclitaxel, carboplatin, etoposide, vinorelbine, and daunorubicin as well as resistance mechanisms of EGFR targeting, have been investigated.

**Results:**

With an elevated dosage of canertinib, a significant decrease in proliferation and increase in apoptosis was observed. The treatment with higher doses of canertinib resulted in a 2-3-fold increase in apoptosis. In the combined treatment, it had been noticed a significant developed apoptotic cell death rather induced by single agent treatment. A significant downregulation of the antiapoptotic protein bcl-2 was exposed by immunocytochemistry investigation. Sensitivity to paclitaxel was also measured and was found to inversely correlate to bcl-2 status.

**Conclusion:**

Proliferation inhibition and apoptosis in MCF-7/TAM-R cells increase with increasing dosage of canertinib. This suggests that canertinib can reverse tamoxifen resistance in breast cancer cells. The antitumor effect of this EGFR-irreversible tyrosine kinase inhibitor provides a rationale for its clinical evaluation in combination with other cytotoxic drugs.

## 1. Introduction

**Figure 1:**
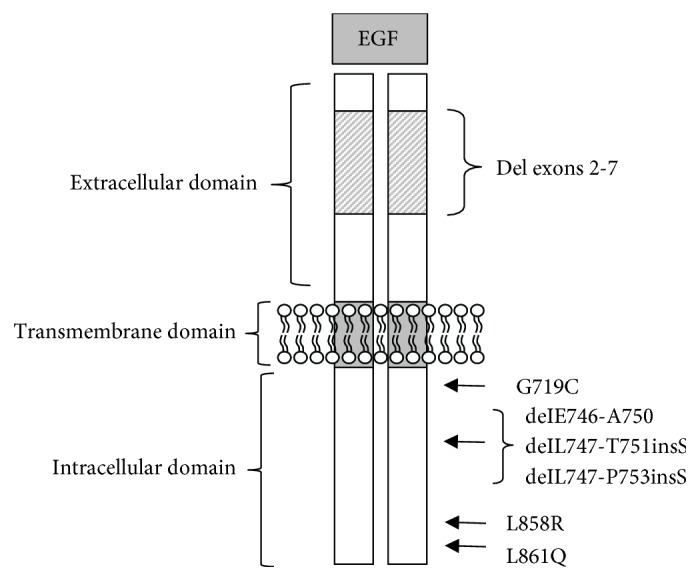
Schematic structure of EGFR with the extra- and intracellular domains.

**Figure 2:**
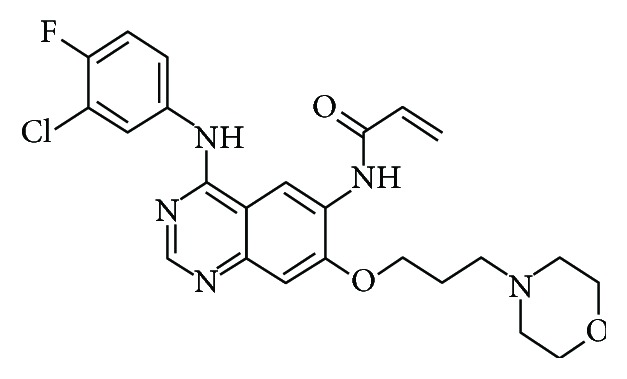
Chemical structure of canertinib (EGFR-irreversible tyrosine kinase inhibitor).

Epidermal growth factor (EGF) was one of the first growth factors discovered which was first isolated by Cohen in 1962 [1]. Moreover, EGF induces cell proliferation or differentiation in mammalian cells through binding to the epidermal growth factor receptor (EGFR) [2]. Further studies elucidated more precise structural details such as the exact amino acid composition [3], the primary structure [4], and the position of the three disulfide bridges shown in Figure 1 [5]. Canertinib was designed as an inhibitor for pan-ErbB tyrosine kinase; it inhibits all four ErbB receptor family members [6] Figure 2.

## 2. Materials and Methods

### 2.1. Materials

Clinical grade canertinib (CI-1033) and cytotoxic drugs (paclitaxel, etoposide, vinorelbine, and daunorubicin) were purchased from Sigma Chemical Co. (Sigma-Aldrich, Egypt). Carboplatin was provided by Sanofi Italia.

#### 2.1.1. Cell Lines and Growth Conditions

The human breast cancer cell line MCF-7 and T47D cells were purchased from VACSERA (Cairo, Egypt); growth conditions of these cells are full RMPl medium supplemented with 5% (*v*/*v*) foetal calf serum, 10 *μ*g/ml penicillin-streptomycin, and 2.5 *μ*g/ml fungizone. Hereafter referred to as RPMl + 5%. To minimize the unwanted oestrogenic effects of phenol red and foetal calf serum, cells were transferred to alternative media prior to the experimentation, containing RMPl medium (w/o phenol red and L-glutamine) supplemented with 5% (*v*/*v*) charcoal stripped foetal calf serum, 4 mM l-glutamine, and 10 *μ*g/ml penicillin-streptomycin.

Establishment of MCF-7(TamR) and T47d (TamR) cells: W + 5% media supplemented with 10^–7^ M 4-hydroxytamoxifen (Sigma-Aldrich, Egypt).

### 2.2. Methods

#### 2.2.1. Western Blot Analysis

Expression and activation levels of several proteins were assayed by Western blot analysis, which was carried out according to the following method (all reagents from Sigma-Aldrich).

Cells were lysed on ice with a lysis solution (50 mM TRIS base, 5 mM EGTA, and 150 mM NaCl, (1% Trion)) containing a cocktail of protease inhibitors (2 mM NaVO4, 50 mM NF, 1 mM PMSF, 20 *μ*M phenylalanine, 10 Mm sodium molybdate, 10 *μ*g/ml leupeptin, and 8 *μ*g/ml aprotinin), the latter two reagents both being added on day of use. The lysates were then centrifuged at 15.300g for 15 minutes at 4°C. The level of EGFR and phosphorylated and unphosphorylated Rb protein was determined by Western blot transfer analysis followed by immunoprobing with respective antibodies and was detected by the enhanced chemiluminescence system.

#### 2.2.2. Cell Viability

Cell viability and sensitivity of the cells to different compounds were determined by 3-(4,5-dimethylthiazol-2-yl)-2,5-diphenyl tetrazolium bromide assay. Cells were plated in duplicate in 96-well cloning tissue culture dishes in specified media. After 48 h, the medium was changed, and Go6976 was added at the desired concentration every 2 days along with fresh medium. After the specified days of growth, the viability of cells was measured and was expressed as a percentage of the untreated control cells grown in the presence of the same concentration of the solvent (DMSO).

### 2.3. Detection of Cellular Apoptosis by Flow Cytometry

Cells were seeded at a density of 40.000 cells/cm^2^ in a 24-well plate and left overnight before being treated with a medium containing 5 mg/ml FITC-dextran (Sigma-Aldrich, Egypt) for 4 hrs. Cells were then washed in situ three times with PBS (37°C) before being trypsinised for 5 minutes in 200 *μ*l trypsin/EDTA (Sigma-Aldrich, Egypt). Following trypsinisation, cells were transferred to centrifuge tubes, pelleted by centrifugation, and resuspended in PBS.

#### 2.3.1. Statistics

All measured data were presented as mean ± standard deviation (± SD) and analyzed using the SPSS program for Windows (SPSS Inc., Chicago, US). ANOVA was performed on each set of data, and a Levene statistic calculated to establish whether variances were equal in sets of data were to be analyzed. Where the Levene statistic was equal to or more than 0.05 and therefore variances were measured to be equal, Dunnett's *t*-test was used to establish whether each treatment had an effect relative to the untreated control group.

## 3. Results

**Figure 3:**
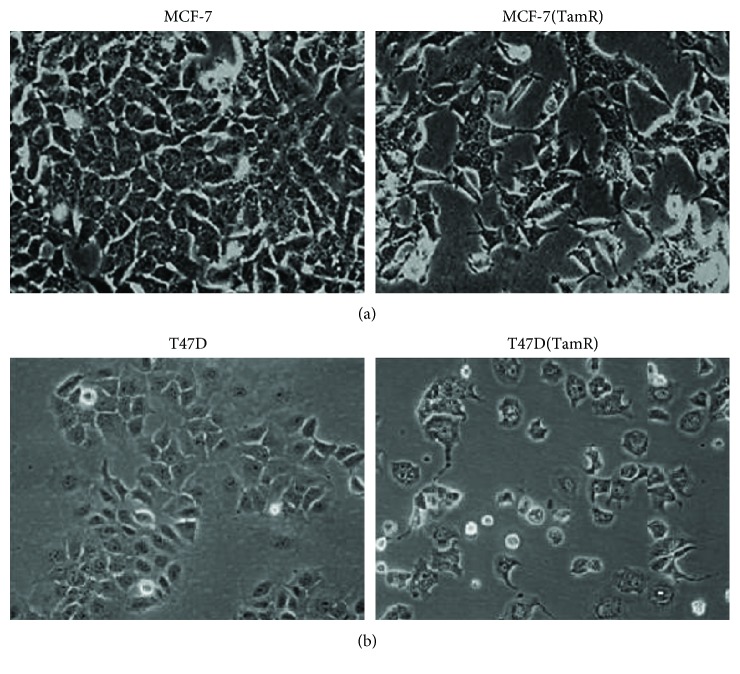
Morphological changes of both cell lines: (a) MCF-7 and its tamoxifen-resistant derivative MCF-7(TamR) cells, (b) T47D and its tamoxifen resistant derivative T47D (TamR).

**Figure 4:**
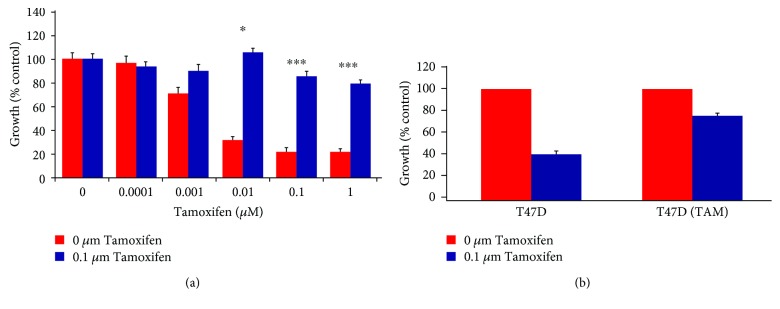
(a) Effect of tamoxifen on the growth of both cell lines MCF-7 (red), and its tamoxifen-resistant derivative MCF-7(TamR) (blue) cells of a 7-day treatment with tamoxifen, added to culture medium and refreshed on day 4. (b) Effect of a 14-day tamoxifen treatment (0.1 *μ*M tamoxifen added to normal growth media) on T47D and its tamoxifen-resistant derivative T47D (TamR) cell line. *N* = 6. *P* values calculated from a paired *t*-test comparing growth inhibition between cell lines at a given concentration of tamoxifen, ∗ indicates *p* < 0.05, *∗∗* indicates *p* < 0.01, *∗∗∗* indicates *p* < 0.001. Error bars indicate standard deviation.

**Figure 5:**
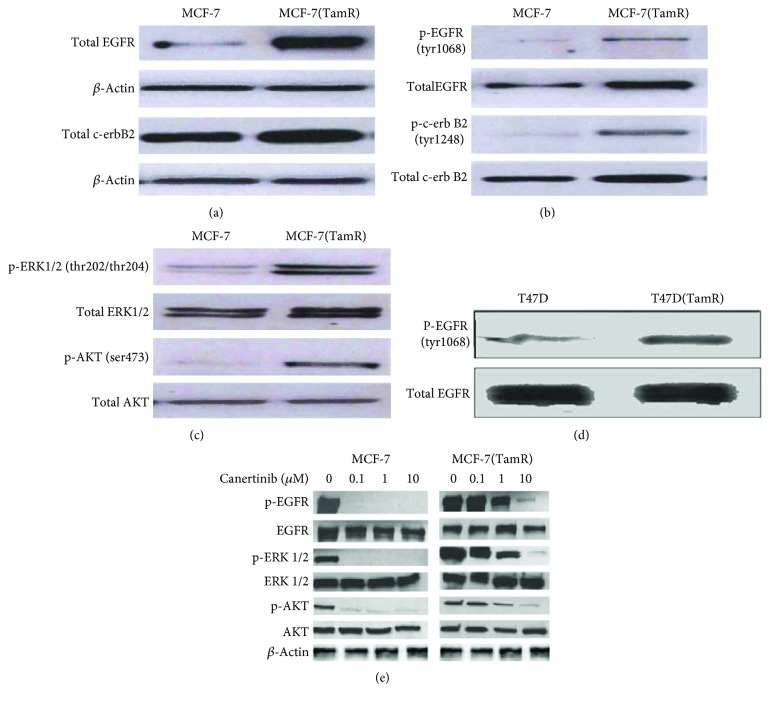
Measuring of EGFR and c-erbB2 expression; activation of ERK1/2 and AKT in MCF-7, T47D, and their tamoxifen-resistant derivative cells MCF-7(TamR) and T47D (TamR) cells, respectively. (a) Changes in levels of total EGFR and c-erbB2 expression by Western blot. (b) Changes in levels of p-EGFR and p-c-erbB2 expression by Western blot. (c) Changes in levels of expression and activation of ERK1/2 and AKT by Western blot. (d) Changes in levels of EGFR expression and activation in t47d cells before and after the development of tamoxifen resistance by Western blot. (e) The inhibitory effects of canertinib on the activation of EGFR, Akt, and ERK1/2 and their encoded protein in both MCF-7 and MCF-7(TamR) cells by Western blot.

**Figure 6:**
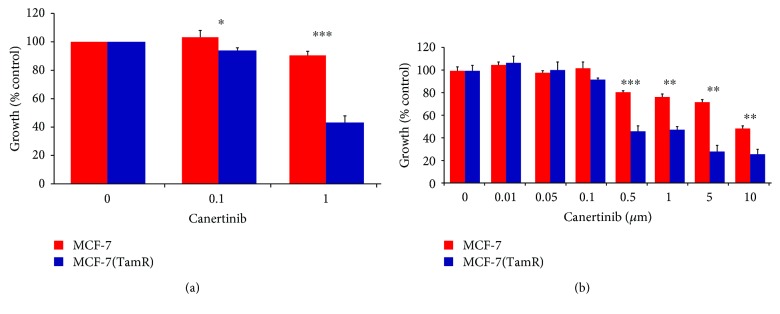
Effect on growth of MCF-7 (red) and MCF-7(TamR) (blue) cells of a 9-day treatment of the EGFR inhibitors (a) canertinib and (b) canertinib added to the cell culture medium and refreshed on day 5 (test comparing growth inhibition between cell lines at a given concentration of EGFR inhibitor). *∗* indicates *p* < 0.05, *∗∗* indicates *p* < 0.01, *∗∗∗* indicates *p* < 0.001. Error bars indicate standard deviation.

**Figure 7:**
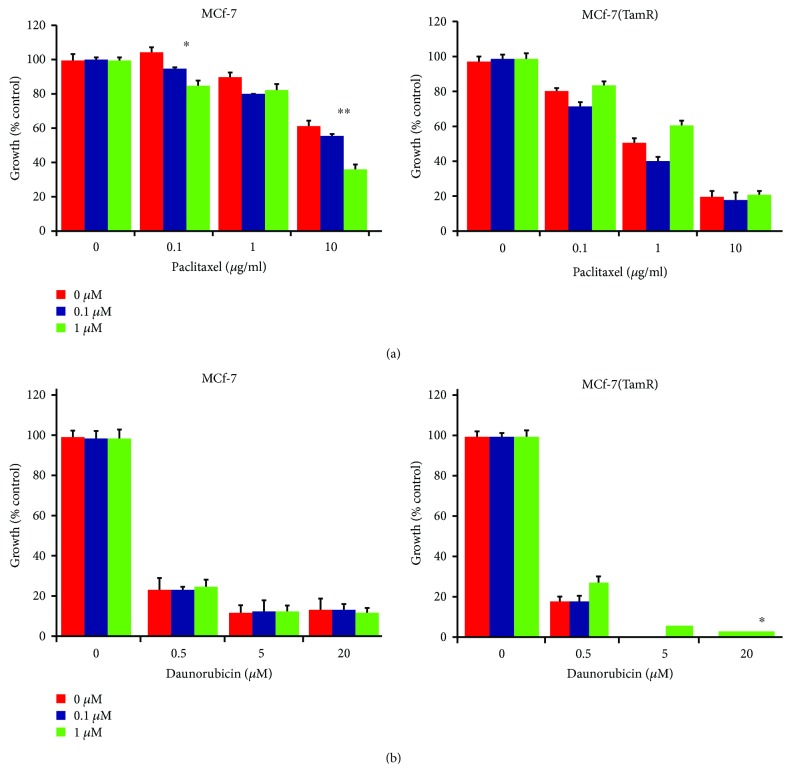
Effect of EGFR inhibition with canertinib treatment on the sensitivity of MCF-7 and MCF-7(TamR) cells to a 48-hour treatment with (a) paclitaxel and (b) daunorubicin. Cells were subjected to a 48-hour cytotoxic treatment with the addition of (red) 0 *μ*M, (blue) 0.1 *μ*M, or (green) 1 *μ*M canertinib before 4 days recovery in the same medium minus the cytotoxic. *P* values calculated from a paired *t*-test comparing growth inhibition between cells untreated with canertinib, both of which were treated with a given concentration of daunorubicin or paclitaxel. ∗ indicates *p* < 0.05 of 0 *μ*M canertinib. Error bars indicate standard deviation.

**Figure 8:**
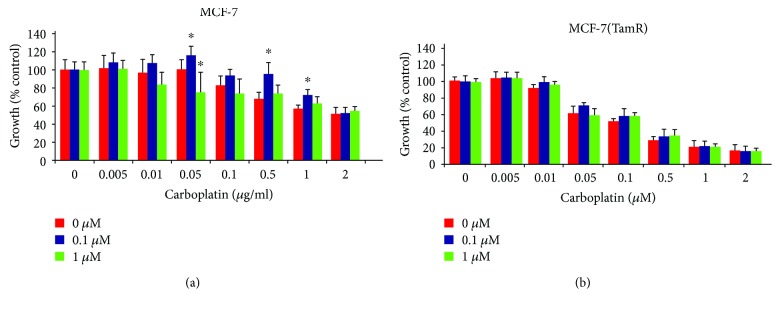
Effect of EGFR inhibition with canertinib treatment on the sensitivity of (a) MCF-7 and (b) MCF-7(TamR) cells to carboplatin. Cells were subjected to a 9-day treatment with carboplatin with the addition of (red) 0 *μ*M, (blue) 0.1 *μ*M, or (green) 1 *μ*M canertinib to the culture medium for the full length of the treatment, refreshed on day 5. *P* values calculated from a paired *t*-test comparing growth inhibition between cells treated with canertinib in addition to a given concentration of paclitaxel. *∗* indicates *p* < 0.05, *∗∗* indicates *p* < 0.01, *∗∗∗* indicates *p* < 0.001 of 0 *μ*M canertinib. Error bars indicate standard deviation.

**Figure 9:**
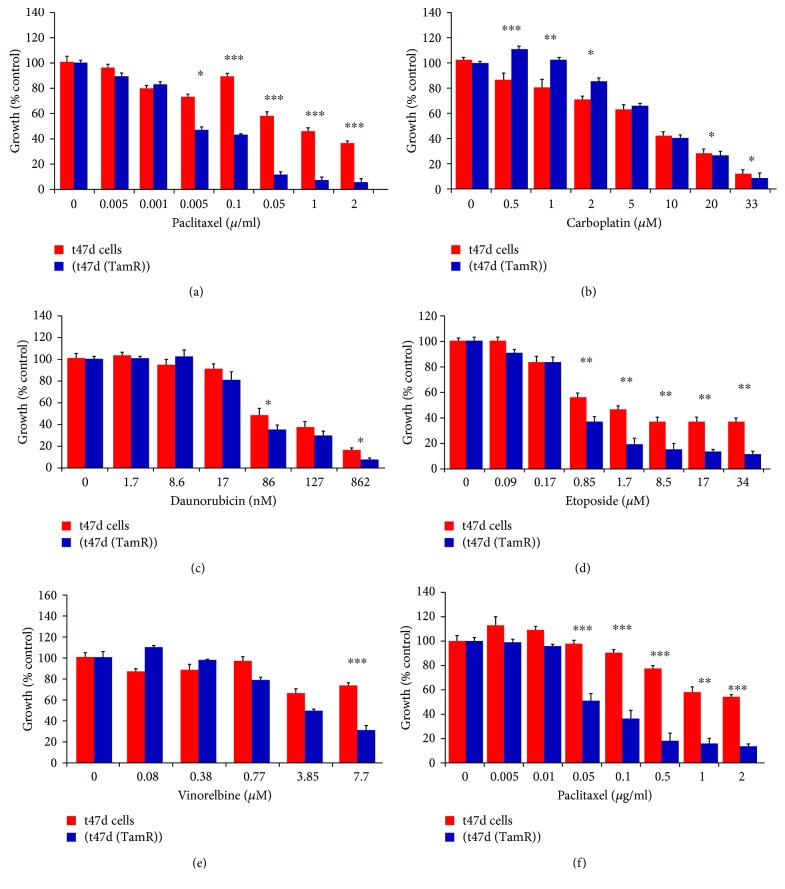
Effect of tamoxifen-resistant breast cancer cells to different cytotoxic agents; cells cultured in media containing paclitaxel for 6 days. (a) Effect on paclitaxel sensitivity, (b) effect on carboplatin sensitivity, (c) effect on daunorubicin sensitivity, (d) effect on etoposide sensitivity, (e) effect on vinorelbine sensitivity, (f) sensitivity of wild-type t47d cells (red) and tamoxifen-resistant t47d cells (t47d (TamR)) (blue) to a 9-day treatment with paclitaxel. Paclitaxel was added directly to the cell culture medium and refreshed on day 5. *N* = 6. *P* values calculated from a paired *t*-test comparing growth inhibition between MCF-7 and MCF-7(TamR) cells caused by a given concentration of cytotoxic agent of interest. *∗* indicates *p* < 0.05, *∗∗* indicates *p* < 0.01, *∗∗∗* indicates *p* < 0.001. Error bars indicate standard deviation.

The tamoxifen-resistant cells developed by long-term culture in 10^–7^ M tamoxifen were examined to ensure that they were able to proliferate in the presence of tamoxifen and that they displayed behavior consistent with previous cell culture models of tamoxifen resistance. A number of changes were observed in both MCF-7 and T47D cells following the development of tamoxifen resistance as the morphology of the cells was significantly altered, with the tamoxifen-resistant MCF-(TamR) and T47D (TamR) cells, respectively, growing in a more scattered fashion and had a more angular appearance which displayed the longer processes previously observed in culture compared to its corresponding wild-type which generally grew in more clearly defined colonies (Figure 3). In addition to their routine growth in 10^–7^ M tamoxifen, MCF-7(TamR) cells also demonstrated the ability to proliferate in a range of concentrations of tamoxifen which were shown to inhibit the growth of wild-type MCF-7 cells (Figure 4), with only a 25% reduction in growth of MCF-7(TamR) cells being observed in a 7-day treatment with 10^–7^ M tamoxifen, compared to 85% reduction in growth of MCF-7 cells. During the development of tamoxifen resistance, levels of expression and activation of several key signaling molecules such as EGFR and AKT are increased, given that increased levels of these proteins, in particularly, EGFR are generally associated with tumors that have increased resistance to cytotoxic drugs. In addition to increased expression of EGFR and c-erbB2, increased activation of these proteins was also observed, with clearly increased levels of phosphorylation of both molecules detected by Western blotting (Figure 5). ERK1/2 activation revealed a clear increased in ERK1/2 phosphorylation, with MCF-7(TamR) cells exhibiting both an increase in staining density and an increase in the number of densely staining cells. Examination of AKT activation also displayed a similar pattern, with a clear increase in staining density following the development of tamoxifen resistance. Western blot analysis confirmed the increase in activation of both ERK1/2 and AKT compared to their total expression levels. The total expression of ERK1/2 seems to increase with the development of tamoxifen resistance, while levels of total AKT seem to remain comparable. MCF-7(TamR) cells became much more sensitive to the growth inhibitory effects of EGFR inhibitors, highlighting their reliance on EGFR signaling for the transmission of proliferative signals. The small molecule inhibitor canertinib, delivered at a concentration of 1 *μ*M over a 48 hours (Figure 6) and 9 days (Figure 7), inhibited the growth of MCF-7(TamR) cells by around 65%, while having no detectable effect on the growth of MCF-7 cells. Much higher concentrations of canertinib (around 10 *μ*M) were able to significantly reduce the growth of both cell lines (Figure 5(e)); however, at all concentrations of canertinib, the growth inhibitory effect was significantly higher in MCF-7(TamR) cells than MCF-7. This is consistent with the observations made in several other studies using various inhibitors of EGFR including gefitinib, lapatinib, erlotinib, LY294002, and Herceptin (Figure 8). Once the expected changes in the activity of signaling molecules in tamoxifen-resistant cells had been confirmed, the effect of these changes on cytotoxic sensitivity was examined. The sensitivity of wild-type, low EGFR expressing MCF-7 cells and tamoxifen resistant, high EGFR expressing MCF-7(TamR) cells to a panel of cytotoxic agents was compared. When treated with paclitaxel there was a marked difference in sensitivity observed between MCF-7 and MCF-7(TamR) cells. Paclitaxel reduced the growth of both cell lines but had a much larger effect on the growth of MCF-7(TamR) cells. Over a 6-day treatment, 0.05 *μ*g/ml paclitaxel reduced the growth rate of MCF-7(TamR) by around 50% while having no significant effect on the growth of MCF-7 cells (Figure 9(a)). Higher concentrations of paclitaxel over 6 days reduced the growth rate of both cell lines but had a much larger effect on MCF-7(TamR) compared to MCF-7 (Figure 9(a)). Treatment with carboplatin also caused a reduction in growth rate of both cell lines, but unlike paclitaxel that had a comparable effect across a wide concentration range. Growth was reduced to a similar extent in both cell lines over the course of a 6-day (Figure 9(b)) treatment period, with MCF-7(TamR) displaying a slightly increased sensitivity at the highest concentrations of carboplatin in both cases. Daunorubicin reduced the growth rate of both MCF-7 and MCF-7(TamR) with a slightly increased effect on MCF-7(TamR) at the highest concentrations (Figure 9(c)). Aside from paclitaxel, the second largest difference in response between MCF-7 and MCF-7(TamR) cells was seen following a 6-day treatment with etoposide with the drug having a significantly larger effect on MCF-7(TamR) cells over a range of concentrations (Figure 9(d)). MCF-7 and MCF-7(TamR) cells displayed broadly similar sensitivity to vinorelbine (Figure 9(e)). The highest concentration of vinorelbine did however have a greater growth reduction effect on MCF-7(TamR) cells in both treatment regimens.

## 4. Discussion

Canertinib is an irreversible inhibitor that binds covalently to specific cysteine residues in the ATP-binding pocket such as cysteine 773 of EGFR, cysteine 784 of ErbB2, and cysteine 778 of ErbB4 thereby blocking the ATP-binding site in the kinase domain of ErbB proteins, preventing their kinase activity and downstream signaling, and additionally, it also prevents transmodulation of ErbB2 [7]. The covalent binding of canertinib results in prolonged suppression of ErbB activity [8]. Since canertinib blocks signaling through all members of the ErbB receptor family, it is more efficient than inhibitors that only prevent signaling from one of the ErbB receptors [9, 10]. Canertinib has been shown to inhibit growth and induce apoptosis in several cancer cell lines [11–13]. In clinical studies, it has been shown to have acceptable side effects. However, in phase II studies canertinib was only able to show modest effects on breast cancer and NSCLC patients [14, 15]. Such drug is able to dephosphorylate p70S6-kinase T389 in a dose-dependent manner as well as to block downstream signaling molecules in all cell lines. Additionally, it induces the apoptosis through an increased expression of the proapoptotic protein BIM (Bcl2-interacting mediator), caspase-3 cleavage, and inhibits proliferation of BCR/ABL-cells resistant to TKIs [16]. It is able to bind not only to the ErbB receptor family but also to intracellular proteins. For instance, the Src kinase family consists of eight members, five of which are mainly expressed in hematopoietic cells, Blk, Hck, Lck, Fyn, and Lyn, where the Lck protein seems to have a stronger binding to canertinib as shown in a protein-binding assay [17].

Initial observation of the MCF-7/MCF-7(TamR) cell culture models revealed that tamoxifen resistance had developed and that the characteristics of interest previously reported by others (Jones et al., 2004; EL-Zarruk and Van den Berg, 1999; Gee et al., 2001; Knowlden et al., 2003) were present [18–21]. These included an increased rate of growth, increased EGFR activity of growth, and increased activation of downstream signaling molecules linked to proliferation and survival. The ability of the increased EGFR signaling to protect MCF-7(TamR) cells from the effects of a panel of cytotoxic drugs was carefully examined, and it was observed that increased levels of EGFR signaling offered cells no significant protection from any of the five drugs studied, and in the case of certain drugs (most clearly with paclitaxel, but also to a lesser extent with other drugs, including etoposide), high EGFR expressing tamoxifen-resistant cells were significantly sensitized to killing with these agents. This observation is completely at odds with all previous studies, which link increased EGFR expression with drug or radioresistant phenotypes. There are many studies which have previously equated an increase in EGFR signaling during tumor development with a resistance to treatment with ionising radiation, for example EGFR signaling was found to be a strong determinant of tumor radioresponse in a panel of murine carcinomas [22] and in human glioblastomas [23], with strong EGFR expression correlating to poor response to radiotherapy in all cases. Consistent with its well-established role in the modulation of cell survival and apoptosis, increased AKT activity has also been linked with cytotoxic resistance. An inducible form of AKT has been shown to protect human myeloid cells from the cytotoxic effects of etoposide and AraC (1-*β*-arabinofuranosylcytosine) [24]. This peculiar sensitivity of tamoxifen-resistant MCF-7 cells in spite of their apparent increase in proliferative and prosurvival signaling was entirely the opposite of what was expected and raises a number of questions concerning the changes that occur in breast cancer cells following the development of tamoxifen resistance. The change in paclitaxel sensitivity looks likely to be caused by a change in signaling molecules or apoptotic machinery lying downstream of the damage event. Bcl-2 looks to be a good candidate, being downregulated in tamoxifen-resistant cells and having a key role in the prevention of apoptosis but attempts to alter levels of bcl-2 and examine the effect on paclitaxel cytotoxicity did not show any clear correlation. It was established that the extent of EGFR signaling appeared to have little effect on the sensitivity of either MCF-7 or MCF-7(TamR) cells to paclitaxel or daunorubicin, as the addition of the EGFR inhibitor, canertinib to cytotoxic treatments had no effect on the toxicity of these drugs to either cell line over a number of different treatment regimens. In addition to this, an alternative model of tamoxifen resistance using t47d cells as the parental cell line displays an identical response to paclitaxel as in the MCF-7-based model, with the tamoxifen-resistant derivative of t47d, (t47d (TamR)) displaying a greatly increased sensitivity, a probable explanation for the inability of increased EGFR levels in tamoxifen-resistant breast cancer to protect against the cytotoxic effects of paclitaxel is that in addition to an increase in EGFR signaling, many other changes in cellular signaling pathways occur during the development of tamoxifen resistance, and that at least one of these changes is responsible for the marked increase in sensitivity to paclitaxel; there are a number of possible candidate pathways that may become altered following the development of tamoxifen resistance and have an effect of paclitaxel sensitivity, including changes in the ability of tamoxifen-resistant cells to take-up or expel drug molecules, differences in the ability of the cells to deal with the damage to DNA caused by cytotoxic drugs, or changes in the apoptotic machinery which may render cells particularly sensitive to attack with certain cytotoxic [25].

## 5. Conclusion

EGFR is an important target for therapeutic intervention in patients with cancer. The present study focused on the inhibitory and antitumor activity of canertinib and provide a rationale for the evaluation of the anticancer activity of this EGFR-specific tyrosine kinase irreversible inhibitor alone and in combination with cytotoxic drugs in different breast cancer cell lines that express functional EGFR, our preliminary results have been reported that the combination of canertinib which is still under phase II clinical studies with different cytotoxic agents significantly inhibits the overexpression of EGFR and markedly increase the sensitivity of different cytotoxic drugs in tamoxifen-resistant breast cancer cells in vitro.

## Data Availability

The data used to support the findings of this study are available from the corresponding author upon request.
